# Effect of Homogenization and Pectin on Chemical, Textural, Antioxidant and Sensory Characteristics of *L. bulgaricus*-Fermented Oat-Based Product

**DOI:** 10.3390/foods14152615

**Published:** 2025-07-25

**Authors:** Dmitrii V. Khrundin, Elena V. Nikitina

**Affiliations:** Department of Meat and Milk Technology, Kazan National Research Technological University, 420015 Kazan, Russia; khrundin@yandex.ru

**Keywords:** oat-fermented base, plant beverages, lactic acid bacteria, fermentation, viscosity, texture, antioxidants, sensory evaluation

## Abstract

The demand for plant-based fermented beverages is being driven by dietary restrictions, health concerns, and environmental concerns. However, the use of plant substrates, such as oats, presents challenges in terms of fermentation and texture formation. The effects of enzymatic hydrolysis, homogenization and the addition of 1% pectin on oat-based beverages fermented with *Lactobacillus delbrueckii* subsp. bulgaricus were evaluated in this study. The samples were evaluated for a number of characteristics, including physicochemical, rheological, antioxidant and sensory properties. After 6 h fermentation, pectin-containing samples showed a statistically significant decrease in pH (to 3.91) and an increase in titratable acidity (to 92 °T). Homogenization and the addition of pectin were found to significantly increase viscosity (by 1.5–2 times) and water-holding capacity (by 2 times) while reducing syneresis by 96%. The antioxidant activity of *L. bulgaricus*-fermented samples increased significantly: the radical scavenging activity (RSA) and OH-radical inhibition increased by 40–60%, depending on the treatment. Extractable polysaccharides (PSs) inhibited lipase and glucosidase by 90% and 85%, respectively; significantly higher inhibition was observed in the fermented and pectin-containing groups. Sensory evaluation showed that the homogenized, pectin-enriched samples (Homog+) scored highest for consistency (4.5 ± 0.2), texture (4.9 ± 0.2), and overall acceptability (4.8 ± 0.2); these scores were all statistically higher than those for the untreated samples. These results suggest that combining enzymatic hydrolysis, homogenization and fermentation with *L. bulgaricus* significantly improves the structural, functional and sensory properties of oat-based beverages, providing a promising approach to producing high-quality, functional non-dairy products.

## 1. Introduction

Traditional fermented milk products contain essential nutrients and bioactive ingredients that are beneficial to human health. Their regular consumption is recommended to the population as part of a healthy diet [1,2,3,4]. On the other hand, dairy products cause a number of nutrition-related side effects such as lactose intolerance and milk protein allergy and thus may be intolerable to an increasing proportion of the population. In addition, animal welfare and environmental issues are important societal drivers for adopting a “free from” diet, which is mainly associated with the consumption of alternative plant-based foods [5,6].

The demand for healthy plant-based foods from consumers around the world is steadily increasing. For example, the global alternative dairy products market size was valued at around USD 38 billion in 2024 (Arizton Advisory and Intelligence 2024, website https://www.fortunebusinessinsights.com/industry-reports/dairy-alternatives-market-100221 accessed on 22 July 2025). Changing dietary behaviors (vegetarian and vegan lifestyles), religious opinions and humanity’s thoughts towards the environment are the major drivers of this process, especially in developed countries [7,8,9].

Nowadays, fermented plant-based products are attracting great interest among consumers [10,11]. Due to their high nutritional value, probiotic, antioxidant and other beneficial properties, such products improve health [12,13,14,15,16]. These products are often seen as an alternative to traditional cow’s milk products [17]. This is especially important for people suffering from lactose intolerance and allergies [18,19].

**Figure 1:**
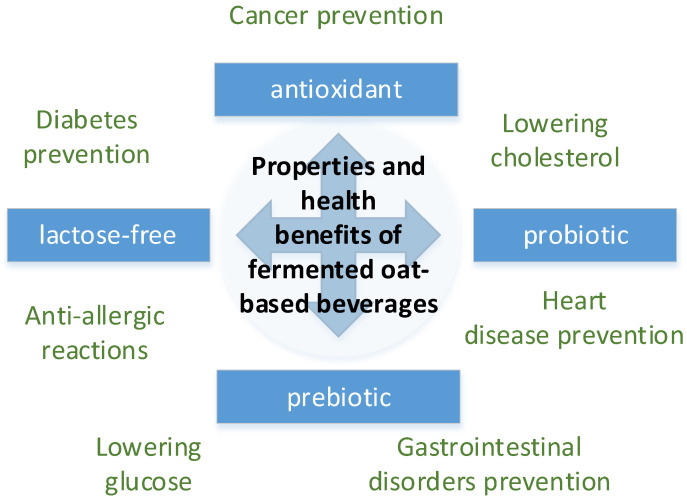
The theoretical basis for the selection of oat-fermented base for plant beverages.

With the growing popularity of plant-based diets, there is a need to develop new products that appeal to the consumer. In essence, botanical sources currently employed in the production of plant-based milk can be classified into five distinct categories: (i) legumes (beans), (ii) nuts, (iii) seeds, (iv) pseudo grains and (v) cereals [20]. Oats are one of the most common cereals. Oats (*Avena sativa*) belong to the Poaceae family and are the sixth most important cereal crop (in terms of production). They are widely grown in many regions, including the European Union, Russia, the United States and Canada, Australia and Brazil [21,22]. Oats are among the most valuable cereal products (Figure 1) in the food market and are considered an exceptional cereal. Oats and their products contain many nutrients including proteins, starch, fats, phenolic compounds, vitamins, and minerals [23].

They play an important role in the prevention of cardiovascular diseases, colorectal cancer, type 2 diabetes mellitus and many other diseases. At the same time, oats are rich in dietary fiber. The main component is β-glucan, which can reduce cholesterol and blood glucose levels after meals. The presence of β-glucan in oats allows one to include oats and products based on them in healthy functional foods; moreover, the beneficial properties of glucans have been proven in chronic diseases [24,25].

The benefits of oat products also lie in the presence of bioactive phytochemicals, avenantramides and avenacosides, which have strong antioxidant and anti-inflammatory effects [25]. Oat flakes have also been found to be suitable for the diet of celiac disease patients [26,27]. This explains the obvious benefits and growing popularity of oat products, especially fermented oat beverages. Fermented oat beverages hold great promise in the market due to their high content of available nutrients, metabolites, and live cultures of lactic acid bacteria. Fermented oat beverages can be consumed not only in pure form. They can be used to make wholesome breakfasts or healthy mid-meals [26].

At the same time, the production of fermented oat beverages remains a difficult task. First, this is due to the large differences between cow’s milk and oat milk as substrates for lactic acid bacteria. The additional preparation of oat raw materials is necessary, for example, the selection of the ratio of the solid to liquid phase, pre-hydrolysis to increase available sugars and more. Second, after fermentation, a structure similar to a fermented milk clot is not formed in the oat beverage. And this is what determines the high flavor and textural properties of traditional yogurt and its popularity [28]. Usually, improvement in the consistency and texture of yogurt has been achieved by increasing the total solids content of the original plant milk or by adding functional ingredients that act as thickeners and stabilizers [29].

These are typically hydrocolloids and each has its own advantages and disadvantages [30]. Different gums are known to be used, which may be suitable for use in some food products as thickeners but may also have adverse effects on the sensory perception of yogurt and its overall quality [28,31]. At present, this remains an open question and further research is required to select a suitable agent within the framework of the concept of healthy eating. The use of pectin seems reasonable to us.

Pectins are widely used as natural stabilizing agents, prebiotics, emulsifiers and thickeners in fermented dairy products [32]. Pectin is a hydrocolloid based on galacturonic acid residues. The intended use of pectin in fermented foods is expanding. Pectin can be a protective agent for lactic acid bacteria [33] and act as an encapsulating agent [34]. The prebiotic function of pectin is well known [33,35]. Moreover, a plant-based fermented beverage composed of prebiotic pectin and probiotic LAB can be symbiotic. This would have a positive impact on the abundance and diversity of the gut microbiota, maintenance of gut health and prevention of chronic diseases [36].

The aims of this study are as follows: (a) to investigate the effect of preliminary hydrolysis by enzyme preparation, the subsequent homogenization of cereal oat raw material and the introduction of pectin on the quality of oat product fermented by *Lactobacillus delbrueckii* ssp. *Bulgaricus* and (b) to evaluate the effect of this treatment on physicochemical, organoleptic, textural–mechanical and antioxidant properties of oat-based drinks fermented by *L. bulgaricus*.

## 2. Materials and Methods

### 2.1. Raw Material and Oat Base Preparation

**Figure 2:**
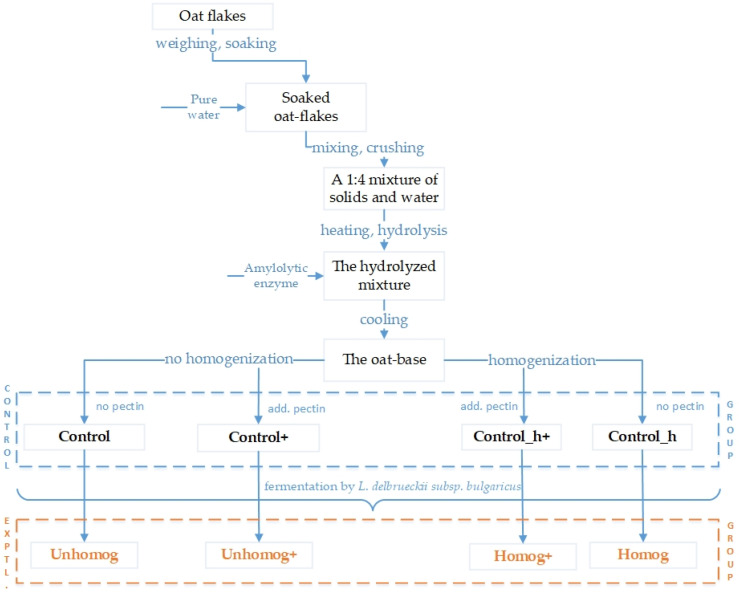
The experimental sample preparation.

Oat flakes were used as the raw source. They were purchased from a local superstore in Kazan city, Russia. Oat flakes were measured and soaked in water for 12 h. The swollen flakes were crushed with water to a homogeneous mixture in a laboratory grinder (“JustBuy,” Qingdao, China). The mixture consists of crushed oat flakes and water in a 1:4 ratio. Then, the mixture was hydrolyzed at a temperature of 60 ± 2 °C for 40 min to improve organoleptic characteristics due to the partial degradation of starch and an increase in the content of free simple sugars using the amylolytic enzyme product (Alfalad™ BN, BioPreparat, Moscow, Russia). To stop hydrolysis, the oat base was heated to 95–98 °C for 5 min. Then, the preparation of the oat base had differences (Figure 2). Then, the mixture was cooled to 37 °C before the addition of the required amounts of starter culture.

### 2.2. Starter Culture

*Lactobacillus delbrueckii* ssp. *bulgaricus* (“Lactosynthesis”, Moscow, Russia) served as a commercial strain for milk dairy products. *L. bulgaricus* was stored in de Man, Rogosa, and Sharpe (MRS) broth (Himedia, Mumbai, India) with 50% glycerol at −80 °C. The cell suspension for inoculation into the oat base was prepared as follows: *L. bulgaricus* cells were inoculated into 5 mL of MRS broth in an amount of 100 µL and were cultured at 37 °C for 24 h. Following centrifugation at 8000 rpm, the cells were resuspended at 37 °C in a sterile 0.85% NaCl solution. The cell concentration was then made the same as 1.5 × 10^8^ CFU/mL (colony forming units/mL), which was the same as 0.5 McFarland units (Densitometer, Biosan, Riga, Latvia). A suspension dosage of 3 mL of suspension per 100 g of the hydrolyzed oat base was used.

### 2.3. Samples

The preparation and description of the samples are presented in Table 1 and Figure 2.

### 2.4. Fermentation Processing

The fermentation of the oat base has been described [37]. The fermentation process was carried out according to two variants: 0–6 h and 0–24 h of fermentation at 40 °C. The physicochemical and rheological parameters and antioxidant properties were determined. A sensory evaluation of the samples was also carried out.

### 2.5. Chemical Assays

Titrable acidity and pH: Titrable acidity was determined by titrating 10 mL of each sample with 0.1 N NaOH, using phenolphthalein as an indicator. An HI 2211-02 (“HANNA Instruments”, Vöhringen, Germany) pH meter was used for the measurement of pH. The the content of protein, fat, solids and total sugars in the samples was determined by near-infrared spectroscopy (“InfraLUM FT-12”, Saint-Petersburg, Russia) with the appropriate software and calibration data. The glucose content of samples was determined by Accu-Chek active GC (“Roche”, Penzberg, Germany).

Total phenolic compound (TPC) assays: The TPCs were analyzed directly in the oat mixture, both before and after the fermentation with *L. bulgaricus*, as well as in the protein-free extracts of all the variants, using the Folin–Ciocalteu reagent, as previously described [37]. The peptide concentration analysis using an O-phthaldialdehyde (OPA) assay has been described previously [38].

The extraction and quantification of extractable polysaccharides (PSs) were performed as described previously [39]. The yield of EPSs was determined by the phenol-sulfuric method [40]. Glucose was used as a calibration standard. The water-soluble components of extractable polysaccharides were used to investigate the antioxidant properties and to analyze the inhibition of glucosidase and lipase.

### 2.6. Analysis of Viscosity and Structural–Mechanical and Textural Parameters

Viscosity and thixotropic properties assays, syneresis analysis and water-holding capacity analysis have been described previously [37]. For the texture profile analysis (TPA), the following factors were determined: hardness (g), cohesiveness, %, gumminess, g, and adhesiveness, g·mm [41].

### 2.7. Antioxidant Assays

The ferric reducing antioxidant power (FRAP) analysis was analyzed according to [42]. Twofold pre-dilution beverage samples were used for the analysis.

The radical scavenging ability (RSA) by the 2,2-Di-phenyl-1-picrylhydrazyl (DPPH) assay was analyzed according to [38]. Tenfold pre-diluted beverage samples were used for analysis. The water-soluble extractable PS was investigated in a similar way, but the initial mixture was diluted fivefold.

OH-free radical scavenging ability was carried out following the procedure described by Sungatullina et al. [42]. Tenfold pre-diluted beverage samples were used for analysis. The water-soluble extractable PS was investigated in a similar way, but the initial mixture was diluted fivefold.

### 2.8. Enzyme Inhibition Assays

For the enzyme inhibition assay, the mixture of water-extractable PSs was diluted fivefold and then analyzed.

α-Glucosidase inhibitory activity: In vitro α-glucosidase inhibitory activity was determined according to the method described by [38] by using p-nitrophenyl-α-D-glucopyranoside (p-NPG) as a substrate and lipase inhibitory activity also occurred. The lipase inhibition activity of EPSs was determined by a method described early [38] using p-nitrophenyl butyrate (NPB) as a substrate.

### 2.9. Method of Sensory Evaluation

Lay panelists in this study were professors and students recruited from among Kazan National Research Technological University and Kazan Technological College to fulfill the evaluation. Participants 18 to 60 years old were invited to participate in this study. Individuals who were lactose-intolerant, pregnant, under 18 or over 60, diabetic, and/or undergoing chemotherapy and individuals who had a tree nut allergy, and/or peanut allergy, and/or soy allergy were not eligible to participate. Ethical approval was also obtained for the publication of the results.

Each expert was seated at a separate desk in the sensory testing area, separate from the preparation room. All participants received a tray of four coded samples at 4 °C in balanced random order. Each tray also included a napkin, a glass of water and the accompanying questionnaire. Before evaluating, participants were instructed to evaluate each sample from left to right and to cleanse their palate with water between each sample. Panelists were also instructed to not speak to other panelists as they completed their questionnaire.

Panelists were asked to rate each sample based on the characteristics of consistency, texture, color, taste, aftertaste, smell, flavor and general acceptability using a five-point hedonic scale (1 = “dislike extremely” to 5 = “like extremely”) with a significance coefficient. These indicators most fully reflect the quality and identification characteristics of plant-based fermented beverages (Table 2).

Thus, the evaluation of plant-based fermented beverages was carried out as follows (Table 3). The panelists evaluated the organoleptic parameters of the product in turn, simultaneously identified the noted inconsistencies and determined the severity of the discrepancy on a 5-point scale: 5—full compliance; 4—single minor inconsistencies; 3—numerous minor inconsistencies; 2—significant inconsistencies; and 1—gross inconsistencies.

The overall assessment of the quality level (Q) of fermented plant-based beverages taking into account the significance coefficient (k) of each organoleptic indicator (B) in points is calculated according to Equation (1):

Q = Ʃ (k·B)(1)

The overall quality score of the evaluated plant-based fermented beverages was calculated as the average of the scores of all panelists. To improve the accuracy of the final result, highly varying estimates were eliminated as outliers. From the remaining number of estimates, the arithmetic mean was calculated again, which was taken as the final score of the sample.

### 2.10. Statistical Analysis

The majority of experiments was carried out in triplicate. Five replicates were performed for antioxidant evaluation. The results were analyzed for statistical significance with a two-way ANOVA by GraphPad Prism 8.0.2 software at a significance level of *p* < 0.05. Data analysis, correlations, and principal component analysis were carried out using Origin8 software version 8.

## 3. Results

### 3.1. Preparation Oat-Fermented Base for Plant Beverages

The first step in the preparation of oat flakes was the removal of impurities, dust particles, etc. Then, we soaked them at a temperature of 25.0 ± 3.0 °C for 8–12 h to swell the proteins, loosen the shells and release intermolecular bonds. The swollen flakes were crushed with the addition of water and hydrolyzed. The hydrolysis process had almost no effect on the properties of the oat base (Table 4).

As expected, the content of dry substances and total sugar (glucose) increased. An increase in protein and fat is probably due to them being released from the native protein–fat–carbohydrate matrix by the action of enzymes and heating. The sensory properties of the oat base improved significantly. The flavor became more harmonious, with pronounced sweetness. The characteristic floury flavor of cereals disappeared. All preparation steps have been described in more detail previously [37]. After this, part of the samples underwent additional processing: they were homogenized for two to three minutes and pectin was added (see Table 1, Figure 1). The processing continued until the pectin had completely dissolved. The suspension was then quickly cooled to the fermentation temperature.

### 3.2. The Oat Base Fermentation Processing

**Figure 3:**
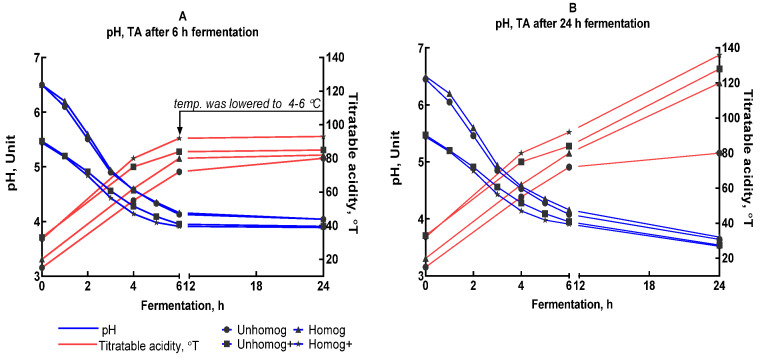

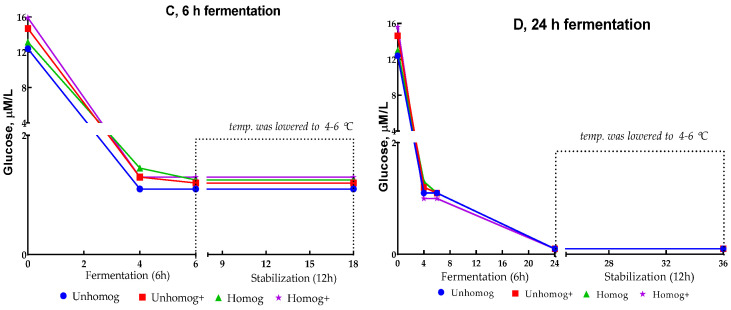
The effects of varying the duration of fermentation and stabilization on changes in titratable acidity, pH (**A**,**B**) and glucose concentration (**C**,**D**). (**A**,**C**) Fermentation for 6 h; (**B**,**D**) fermentation for 24 h.

Two fermentation durations were evaluated: 0–6 h and 0–24 h of fermentation. The intensity of fermentation was determined by changing the pH, titratable acidity and glucose level (Figure 3A–D).

At the initial stage (two hours), there were no significant changes in the parameters. This is probably due to the adaptation of LAB, the heating of the samples to the cultivation temperature, and so on. After four hours, clear signs of fermentation appeared, such as a sour taste and faint odor. There was an active accumulation of acid, as demonstrated by a decline in pH, and the glucose level in the samples also diminished significantly.

The most significant changes were observed in the Unhomog+ and Homog+ samples: 75 °T (pH = 4.56) and 80 °T (pH = 4.14). At 6 h, fermentation increased: the smell and taste became noticeable. The titrated acidity (pH) reached values from 72 °T (4.13)—Unhomog—to 92 °T (3.91)—Homog+. At this step, some of the samples were placed in a freezer to stop fermentation. And then they were placed into the refrigerator to stabilize the system. The fermentation of the remaining samples was continued for up to 24 h in order to determine the maximum allowable level of titrated acidity (pH). Fermentation was intense, and the glucose level was very low. As a result, the titrated acidity (pH) reached values from 80 °T (3.69)—Unhomog—to 136 °T (3.52)—Homog+.

Thus, irrespective of the presence of pectin or pre-homogenization, L. bulgaricus fermented the oat base efficiently. After 24 h of fermentation, the samples have a sour taste and a pungent, unpleasant odor. The experiments revealed that six hours of fermentation was the perfect amount of time to achieve an oat base with the ideal acidity and a delicious flavor. This finding led to the decision to extend the fermentation time to six hours in subsequent experiments.

### 3.3. Properties of Oat-Fermented Base After 6 h of Fermentation

#### 3.3.1. Chemical Composition Changes

**Figure 4:**
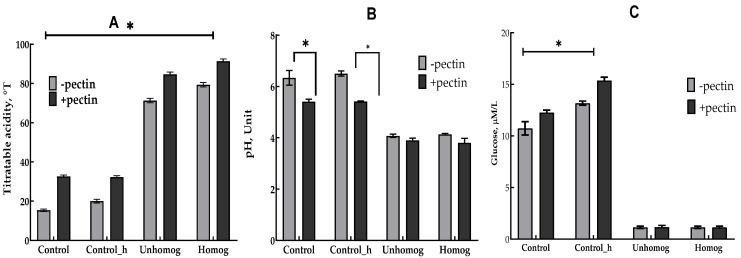
Effect of *L. bulgaricus* fermentation and pectin on titratable acidity (**A**), pH (**B**), and absolute glucose concentration (**C**). Asterisks indicate statistically significant differences between variants without pectin and with pectin according to non-parametric one-way analysis of variance (Kruskal–Wallis) test, *p* < 0.05.

The results of the study showed a high adaptation of LAB to the oat base. Samples without pectin addition had titratable acidity 72–84 °T and pH 4.13–3.95 after 6 h of fermentation (Figure 4A,B). The titratable acidity increased and pH decreased by adding pectin to the oat base. The utilization of carbohydrates, especially glucose, correlated with the level of acid formation. By adding pectin, the glucose content of the oat base was increased (Figure 4C).

A composition analysis of the samples showed comparable amounts of protein, fat and dry matter regardless of the treatment. The samples Unhomog+ and Homog+ contained more dry matter, which is explained by the presence of pectin (Table 5).

#### 3.3.2. Structural and Textural Properties

For fermented dairy products and their plant-based counterparts, which are complex systems (emulsions and suspensions), the viscosity, water-holding capacity (WHC) and tendency to separate (syneresis) are crucial factors for their stability and consumer appeal.

**Figure 5:**
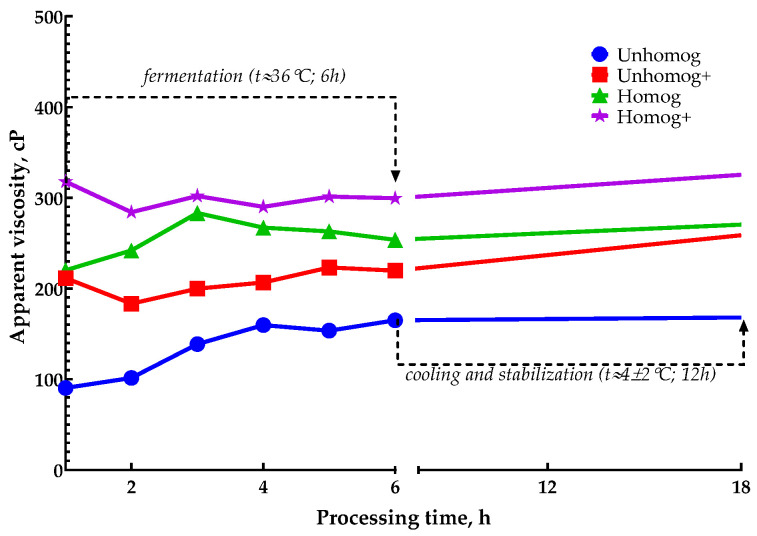
Viscosity changes during 6 h *L. bulgaricus* fermentation and after stabilization at 4 °C.

The change in the viscosity of the samples had the same character (Figure 5). In the range from 0 to 6 h, the viscosity changed slightly with a tendency to increase. During the stabilization process, the viscosity increased by an average of 30–50 cP, with the exception of the Unhomog sample.

As expected, the increase in the viscosity of samples with pectin is due to its swelling ability, which is widely used in food processing. The lowest viscosity value was in Unhomog, while the highest values were in Homog+ and the viscosity loss (L_ƞ_) was reversed (Table 6).

After adding pectin to the oat base, syneresis decreased and WHC increased. The fermented sample had improved syneresis and WHC values, especially the homogenized ones. The Unhomog had highest water loss (Syn = 24.3%) despite high WHC (47.5%); this ratio suggests that the sample’s matrix structure is not robust and may be prone to delamination. The Homog+ had the lowest water loss (Syn = 0.5%) despite high WHC (98.0%). This is likely due to a reduction in particle size, an expansion of the wetting surface area, and the stabilization of the whole system.

The results of texture profile analysis are presented in Table 7. Changes in the texture of the oat-fermented base for the plant beverage are due to fermentation (increasing the maximum force and hardness). The addition of pectin increased the residual force after relaxation and it increased the adhesive force. Viscoelastic properties were enhanced too. Textural properties of Homog and Homog+ were changed the most. Probably due to the homogeneous structure, a more stable spatial matrix was formed (correlated with the data in Table 6).

The data obtained are of great practical importance, since gumminess and adhesion affect sensory perception: the ability of the product to envelop the oral cavity and adhere to the tongue and palate. The prolonged exposure of the receptors allows for a better perception of taste, smell, aroma, and aftertaste.

#### 3.3.3. Sensory Evaluation

An assessment of organoleptic quality indicators of food products, especially new ones, is required the most [43,44,45,46]. It gives statistically correct results and does not require any special equipment; the analyzers are the human sense organs. The sensory assessment data are summarized in Table 8.

Appearance: There was no significant overall difference amongst participants’ perception of appearance for all samples. All samples had the acceptable appearance expected for this kind of product.

Color: There was no significant overall difference amongst participants’ perception of color for all samples. All samples had an acceptable appearance characteristic of oats and oat products.

Consistency and texture: There was a significant difference between participants’ consistency and texture perception between the fermented samples. Intra-sample comparisons reveal that there was a significant difference between samples with pectin and without it. Participants noted a more homogeneous and tightened consistency of such samples. The texture was also more enveloping. It gave a pleasant sensation on the tongue and in the mouth. The mean score for the consistency (texture) of Homog+ was the highest—4.5 ± 0.2 (4.9 ± 0.2)—and Unhomog scored the lowest—3.4 ± 0.2 (3.2 ± 0.2).

Taste and aftertaste: There was a significant overall difference in participants’ perception of the taste and aftertaste of all samples. The most developed taste and aftertaste were in Unhomog+ and Homog+ (pronounced and pleasant) and the least developed in the oat base (not pronounced and weak, no aftertaste). The mean score for taste and aftertaste among the fermented samples was the highest for Homog+ with 4.9 ± 0.2 (4.4 ± 0.2), and Unhomog scored the lowest with 3.4 ± 0.2 (2.2 ± 0.2).

Smell and flavor: There was a significant overall difference in participants’ perception of the smell and flavor of all samples. The most developed taste and aftertaste were in Unhomog+ and Homog+ (pronounced and pleasant) and the least developed in Unhomog (not pronounced and weak and no aftertaste).

General acceptability: The participants pointed out the high sensory perception of all fermented samples. However, the intra-sample comparison revealed that there was a significant difference between Unhomog and Homog, regardless of the presence of pectin. Unhomog had a more heterogeneous consistency with pronounced individual particles of flakes, and Homog was smoother and more homogeneous. Pectin both expectedly increased viscosity and had a greater effect on texture, making it more elastic and enveloping. The mean score for general acceptability among the fermented samples was the highest for the sample Homog+ (4.8 ± 0.2), and Unhomog scored the lowest (3.4 ± 0.2).

### 3.4. Total Phenol-Containing Compounds and Antioxidant Properties

**Figure 6:**
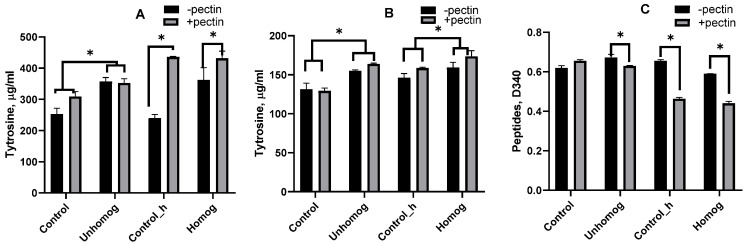
Effect of homogenization and pectin added to concentration on total phenol-containing compounds (TPCs) ((**A**)—product; (**B**)—protein-free extract) and peptides in protein-free extract (**C**) in *L. bulgaricus*-fermented oat base. Control—unfermented oat base, Control_h—unfermented homogenized oat base, Unhomog—*L. bulgaricus*-fermented oat base, Homog—*L. bulgaricus*-fermented homogenized oat base. Asterisks indicate statistically significant differences between variants without pectin (control) and with pectin according to non-parametric one-way analysis of variance (Kruskal–Wallis) test, *p* < 0.05.

The raw materials of vegetable origin are a source of polyphenolic compounds and these compounds are modified under the action of LAB. In the controls without pectin, the amount of total phenol-containing compounds (TPCs) was approximately at the same level, both with and without homogenization (Figure 6A). The addition of pectin to the controls resulted in an increase in the TPC content due to the presence of TPC in pectin. After *L. bulgaricus* fermentation, the amount of TPC in the product increased significantly, which is probably a consequence of the release of phenolic compounds from the plant oat matrix and pectin under the action of the enzyme systems of the lactic acid bacterium.

The analysis of low-molecular-weight TPC in protein-free extract (PFE) revealed minimal or no effect on this amount from the addition of pectin. The amount of TPC in PFE was increased by the homogenization of the oat base. Furthermore, the amount of TPCs increased even more following the fermentation of *L. bulgaricus* (Figure 6B). The bioavailability of TPCs is increased by the action of *L. bulgaricus* enzyme systems, which is a positive outcome since TPCs are a group of substances with antioxidant potential.

The amount of peptides in the PFE of the Control_h sample was slightly higher than in the control sample, probably due to the destruction of plant components during homogenization (Figure 6C). Adding pectin to the control sample decreased the amount of free peptides, probably due to the sorption of low-molecular-weight protein compounds by the pectin. Following *L. bulgaricus* fermentation, the quantity of free peptides increased in the oat base, even without homogenization or pectin. The amount of peptides decreased in the homogenized sample without pectin, possibly indicating a more active peptide metabolism of bacteria. Homogenization increases the bioavailability of protein components, especially low-molecular-weight ones.

**Figure 7:**
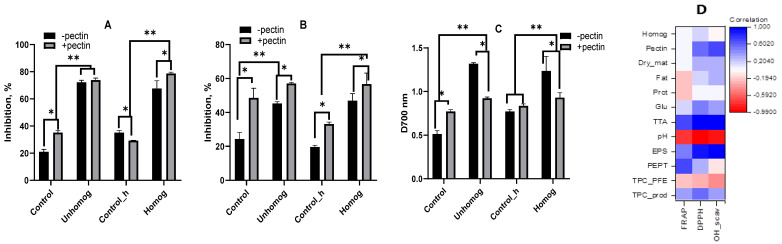
Antioxidant activity of *L. bulgaricus*-fermented oat base: radical scavenging ability (**A**), OH-free radical scavenging ability (**B**), ferric reducing antioxidant power (**C**), and correlation (**D**); Control—unfermented oat base, Control_h—unfermented homogenized oat base, Unhomog—*L. bulgaricus*-fermented oat base, Homog—*L. bulgaricus*-fermented homogenized oat base. Asterisks indicate statistically significant differences between variants without pectin (control) and with pectin according to non-parametric one-way analysis of variance (Kruskal–Wallis) test, *p* < 0.05. “*”—indicates differences between versions with and without pectin. “**”—indicates differences between control (before fermentation) and after fermentation by *L. bulgaricus*.

The impact of homogenization and the presence of pectin on antioxidant properties in oat base was examined in three tests: radical scavenging activity (RSA), OH-free radical scavenging ability (OH-SA) and ferric reducing antioxidant power (FRAP) (Figure 7).

In the control without homogenization (control), RSA was lower than in the control with homogenization (Control_h), which indicates the release of components with free radical-binding capacity from the oat grain. The presence of pectin in the control variant led to an increase in RSA, while RSA decreased in the Control_h variant. This could be a result of pectin’s ability to interact with and bind to low-molecular-weight components. The fermentation of the oat base of *L. bulgaricus* resulted in a significant increase in RSA in all samples.

It was shown by OH-SA testing (Figure 7B) that an increase in activity was not led to by homogenization, whereas hydroxyl radical binding activity was significantly increased by the use of pectin. So, like with the RSA, after six hours of *L. bulgaricus* fermentation, OH-SA went up in all the samples.

Reducing activity is characterized by the ability to convert Fe^3+^ into the Fe^2+^ form. FRAP was found to be higher in the homogenized control than in the non-homogenized control (see Figure 7C), probably due to the release of carbohydrate-reducing components from starch. The addition of pectin to the controls resulted in a slight increase in FRAP. The FRAP levels of samples fermented with *L. bulgaricus* increased, particularly in the variants devoid of pectin (black bars).

An analysis of the correlations revealed that the homogenization of the oat base is not a key factor in the manifestation of the antioxidant properties; the correlation coefficient is approximately 0.2 or lower (Figure 7D). The formation of the antioxidant properties of the oat base is significantly influenced by the use of pectin (correlation 0.5–0.6). The manifestation of the antioxidant properties of RSA and OH-SA positively correlates with the level of lactic acid accumulation, which is an indicator of fermentation by lactic acid bacteria. Additionally, the RSA and OH-SA of the fermented oat base depend on extractable polysaccharides and TPCs, which we will discuss below. Furthermore, FRAP is positively affected by the accumulation of peptides in the product.

### 3.5. Extractable Polysaccharides (PSs) and Their Antioxidant Properties

Lactic acid bacteria can synthesize exopolysaccharides, but it is difficult to analyze them under plant beverage conditions because starch and non-starch polysaccharides of plant origin are present. When it comes to plant-fermented products, it is more accurate to talk about the total amount of extractable polysaccharides (PSs) from both plant and bacterial sources.

**Figure 8:**
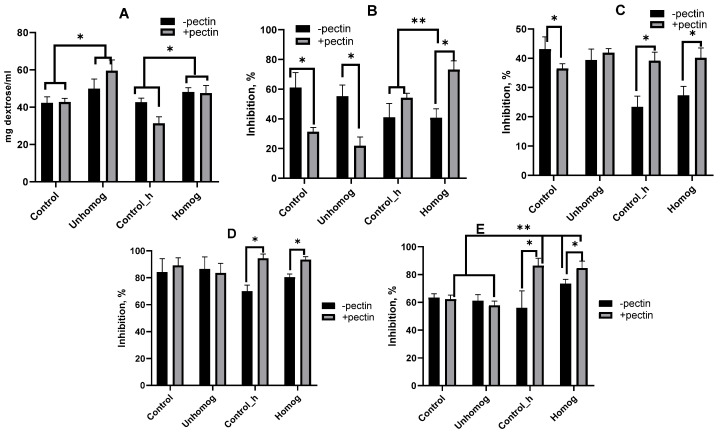
Total amount of extractable polysaccharides (PSs) (**A**), radical scavenging ability (**B**), OH-free radical scavenging ability (**C**), lipase inhibition (**D**), glucosidase inhibition (**E**) of EPS extractable from *L. bulgaricus*-fermented oat base. Control—unfermented oat base, Control_h—unfermented homogenized oat base, Unhomog—*L. bulgaricus*-fermented oat base, Homog—*L. bulgaricus*-fermented homogenized oat base. Asterisks indicate statistically significant differences between variants without pectin (control) and with pectin according to non-parametric one-way analysis of variance (Kruskal–Wallis) test, *p* < 0.05. “*”—indicates differences between versions with and without pectin. “**”—indicates differences between variants without homogenization and with homogenization.

The amount of extractable PS in the control variants was unaffected by homogenization without the pre-fermentation of *L. bulgaricus* (Figure 8A). An alternative scenario was identified when pectin was included. With regard to the non-homogenized control (control), the incorporation of pectin had no impact on the extractability of the quantity of extractable PS. Conversely, in the homogenized variant (Control_h), a reduction in the amount of extractable PS was detected. Fermentation by *L. bulgaricus* led to an increase in the amount of PS in the samples, especially in the variants with pectin. The process of homogenization did not affect the increase in extractable PS in the variants without pectin. In both variants, the amount of extractable PS increased by about 5 mg/mL. Applying pectin increased the amount of extractable PS by 16–17 mg/mL compared to the initial level of the controls after fermentation.

The antioxidant properties of lactic acid bacteria exopolysaccharides have been reported in numerous studies [38,47]. The method of extraction results in a substance with a high content of plant polysaccharides. This means that it is not possible to speak about the exclusive role of bacterial EPSs alone in the tests. The addition of pectin was found to decrease the radical scavenging ability (RSA) of EPSs in variants without homogenization (Figure 8B). Furthermore, the RSA of polysaccharides did not increase after *L. bulgaricus* fermentation. In homogenized variants, adding pectin increased the RSA of EPS, which was even higher in EPS isolated after fermentation by *L. bulgaricus*. A similar trend was observed when testing the OH-free radical scavenging ability of EPS (Figure 8C).

The activity of lipase and glucosidase can be inhibited by exopolysaccharides produced by lactic acid bacteria [48,49]. In the oat-based variants that were not homogenized, the percentage of lipase inhibition was high at around 90%, and adding pectin or *L. bulgaricus* fermentation did not affect this percentage (Figure 8D). For homogenized samples without pectin, the percentage of lipase inhibition was lower than for the non-homogenized variants. Adding pectin resulted in extractable PSs inhibiting lipase more effectively than PSs from non-homogenized samples.

As with lipase, extractable PSs from non-homogenized samples (control and Unhomog) inhibited glucosidase to the same extent (approximately 60%) (Figure 8E). However, extractable PSs from the homogenized sample without pectin only inhibited glucosidase by 56%, a figure which increased to 73% with the addition of pectin. Fermentation with *L. bulgaricus* led to an increase in inhibition to 83–85% in variants with and without pectin.

## 4. Discussion

Oat products are growing in popularity because of their ingredients and the positive effects they have on health [50,51,52,53]. The elevated fiber composition (notably β-glucans) enhances cardiac and vascular functionality [51,54,55]. The presence of antioxidants, vitamins and minerals keeps you healthy and boosts your immune system [51,52]. Oat products can be recommended as part of a healthy diet thanks to their high protein and amino acid content and low glycemic index [3,24]. They have the ability to envelop the walls of the stomach with a protective film and reduce the acidity of gastric juice, which is important for problems with the gastrointestinal tract [54,55], and they reduce cholesterol levels [56,57].

The results of this study demonstrate the significance of enzymatic and mechanical pretreatment in enhancing the properties of oat-based systems for fermentation. Prior to the commencement of treatment, the oat suspension exhibited clear phase separation and unmeasurable viscosity. Following the processes of hydrolysis and homogenization, the suspension demonstrated enhanced stability and increased viscosity (100–114 cP), thus signifying the efficacy of the structurally modifying alterations undergone by the matrix. The positive impact of homogenization on the processing of plant products is evidenced by a number of studies [58,59], and to make milk, increasing stability and improving sensory characteristics are essential [60].

The process of extracting useful components from cereal raw materials involves the following steps: digestion, enzymatic hydrolysis and homogenization [61,62,63]. Enzymatic hydrolysis significantly increased the glucose content of the oat base (up to 15–17 μM/L), indicating improved carbohydrate availability for fermentation.

Mechanical homogenization increased the degree of homogeneity of the samples, especially in the Homog and Homog+ groups. The impact of lactic acid fermentation was found to be a significant factor, with a notable enhancement in the observed effects of viscosity. The likely mechanism is associated with the formation of an enhanced protein–polysaccharide matrix, a consequence of two phenomena: firstly, decreased particle size, and secondly, increased interaction surface area [43]. Pectin contributes to this stability by exerting a gelling and water-binding effect, which contributes to the observed improvement in viscosity and sample integrity [32,58,64].

As shown in Figure 5, viscosity was more strongly affected by homogenization than by enzymatic treatment. At the same time, the addition of pectin had a more pronounced effect on water-holding capacity and syneresis (Table 6). These results confirm that the combination of enzymatic hydrolysis, homogenization and pectin addition synergistically improves the functional and rheological characteristics of oat-based systems intended for fermentation.

At the same time, the potential of the oat base is far from exhausted. The final product is significantly enriched and its functional properties are enhanced by the enzymatic hydrolysis of oat raw material and fermentation using lactic acid bacteria [4,14,65]. The investigation revealed that *L. bulgaricus* can adapt to oat substrate, particularly following pre-treatment. The titratable acidity values increased three to fivefold after six hours of fermentation, especially in the Homog+ sample, where they reached 90 °T. The enhancement in acidity concomitantly elevates the sensory perception of the product. The flavor and odor became more pronounced, and the consistency more homogeneous and denser. Additionally, the fermentation of oat bases by lactic acid bacteria significantly contributes to the flavor and aroma of oat products. Many authors emphasize this role by using different starters to create different flavor and aroma profiles for the product [66,67].

Many studies in the field of plant beverages highlight the common disadvantages of such products, such as their tendency to separate and their insufficiently viscous texture [68,69,70,71]. These factors significantly impact consumer preferences [46,72,73]. We proposed the use of pectin to correct rheological and textural parameters. The use of pectin is widespread in food production, where it serves the roles of thickener and stabilizer [32,34,69]. There are a number of positive properties that are associated with pectin [74,75]. A thorough investigation revealed that pectin significantly enhanced the rheological and functional characteristics of the oat base.

**Figure 9:**
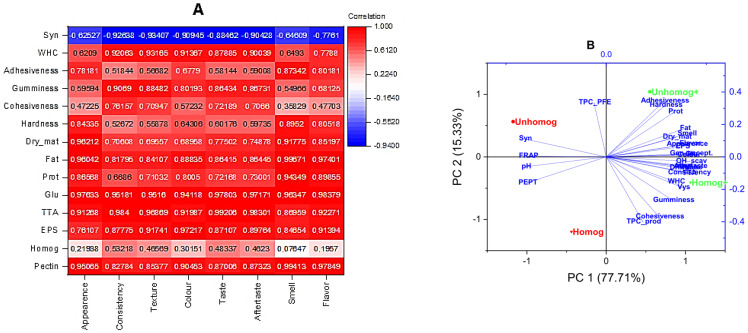
The heatmap correlation of sensory profile and chemical/texture parameters (**A**) of oat-based beverages fermented by *L. bulgaricus* with/without pectin and the principal component analysis of beverages (**B**).

An analysis of TPA data revealed that pectin significantly enhances the adhesion and cohesion of samples, which improves sensory perception. This is confirmed by the analysis of the heat map of the sensory profile and chemo-textural parameters (Figure 9A), which shows a positive correlation between the organoleptic parameters and the presence of pectin (consistency: 0.83; texture: 0.85).

The relationship between the sensory characteristics of food products and their rheological properties is being investigated by researchers. The formation of consumer preferences was the focus of the study by Castro et al., who investigated the impact of the fermentation process and the type of LAB culture [76]. The need to combine sensory analysis methods with modern instrumental techniques and mathematical models to improve the objectivity of the results is confirmed by Rodrigues et al. [44]. A correlation was found between TPA and the sensory characteristics of fruit texture [77].

This will enable sensory expectations to be predicted and adjusted during the production process. This is in contrast to tasting, which involves analyzing the product.

The analysis of the experimental data showed a high positive correlation between sensory evaluation and instrumental indices: consistency—gumminess (0.91), texture—gumminess (0.88), consistency—cohesiveness (0.76), texture—cohesiveness (0.71), taste—gumminess (0.86), and aftertaste—gumminess (0.87).

The consistency, texture and flavor of the product are also significantly improved in the presence of pectin. This dependence is important because it increases our understanding of how components influence the sensory perception of the product. The interconnection between subjective evaluation (enveloping properties, prolonged contact with the mouth and tongue, and a velvety texture) and the outcomes of instrumental assessments is demonstrated. The relationship between sensory profile, texture and processing parameters was also revealed to have a significant correlation with more than 10 controlled parameters by principal component analysis (Figure 9B), especially with those containing pectin, as shown by the homogenization of the samples. Kaur et al. positively evaluate homogenization as a processing method for vegetable raw materials, as it increases the availability of their components and improves flavor and aroma [58]. A significant correlation was found between homogenization and the rheological properties of fruit-containing products by Salehi et al. [59]. Moreover, another study found that homogenization had a positive effect on the stability of herbal beverages, increasing their viscosity and improving their overall quality [78].

Thus our research confirms that the textural and functional characteristics of the oat-based system developed in this study play a key role in its technological and nutritional value. Our results indicate that viscosity is a key factor affecting flavor and mouthfeel. Not only glucan is involved in flavor formation; structural changes introduced during fermentation play a positive role. Of course, the beneficial role of β-glucan, known for its rheological and physiological functions [79], is complemented by microorganisms, in particular *Lactobacillus bulgaricus*, which synthesizes exopolysaccharides (EPSs) during fermentation. The ability of *L. bulgaricus* to produce EPSs on cereal substrates plays an important role in improving the texture and stability of the final product [80], previously demonstrated in milk [41]. The presence of EPS-producing LAB in the fermentation matrix is consistent with a broader trend in food biotechnology, where microbial EPSs are valued as in situ bio-thickeners that improve texture and flavor without synthetic additives [81]. A number of studies have demonstrated the positive role of lactobacilli in the fermentation of herbal beverages in terms of texture, viscosity and sensory qualities [82,83,84,85].

Our data show that this microbial contribution is not limited to technological aspects; it also extends to biofunctionality. Both β-glucans and EPSs have been recognized for their prebiotic effects, making their combination in a single product particularly relevant in the context of functional nutrition [86]. An important observation was the decreased extractability of polysaccharides in systems where pectin and homogenization were combined. Rather than being a drawback, this points to potential encapsulation and binding mechanisms—such as ionotropic gelation and electrostatic interactions—that could contribute to a controlled release of bioactives and improved stability [87]. These properties have been described for pectins [88,89] and are an example of electrostatic binding and encapsulation due to anionically charged carbohydrates. This aspect underscores the need to consider not only yield but also the functional matrix interactions when designing plant-based products.

The biological activity of plant polysaccharides is often low, failing to meet the needs of those seeking to maximize the benefits of prebiotic products. A number of authors have reported the antioxidant properties of plant polysaccharides [90,91]. Considering the enzymes and organic acids produced by *Lactobacillus*, fermentation by this bacterium can increase the extraction efficiency of polysaccharides [92] and affect their structure and biological activity [93]. We discovered that the antioxidant activity of the fermented oat product and the extractable polysaccharides increased when we used *L. bulgaricus* for the fermentation of oat base starter culture. A number of works obtained similar data when they used products of oat grain processing and lactic acid bacteria [94,95]. The increase in antioxidant properties of plant products after fermentation by lactic acid bacteria is due to the increase in the bioavailability of plant components such as phenol-containing substances and flavonoids, which have antioxidant properties. Research shows that fermentation by *L. bulgaricus* leads to an increase in phenol-containing compounds, which contributes to the product’s antioxidant potential. Wen et al. 2020 [96] found that fermenting lychee juice with Lactobacillus casei increased the content of phenols, flavones and exopolysaccharides, thereby improving its immune-modulating properties and ability to alter the gut microbiota. It was hypothesized that the fermentation of *Lactobacillus plantarum* in Chinese dwarf cherry juice would result in an increase in phenolic compounds, which have been shown to have a beneficial effect on the health of laboratory animals [97].

Our findings suggest that LAB fermentation, particularly with *L. bulgaricus*, is an effective way to improve the sensory and rheological properties of cereal-based beverages, as well as their functional bioactivities, such as antioxidant, anti-diabetic and cholesterol-lowering effects [98,99,100]. The results of our study showed that fermented extracts were more effective at inhibiting lipase and glucosidase. This suggests that there is a complex relationship between microbial activity and how substrates are transformed, highlighting the importance of metabolic interplay in these processes [101]. This is comparable to the results seen in rice-based systems fermented with *Lactobacillus pentosus*, where lipase inhibition rose significantly after fermentation [102].

Our results show that combining mechanical processing (e.g., homogenization), natural gelling agents (e.g., pectin) and strategic microbial fermentation (e.g., via *L. bulgaricus*) can improve the technological and health-related qualities of oat-based beverages. In addition to product optimization, these methods are an eco-friendly and secure approach to enhancing the nutritional content of plant-based foods, which is key to the creation of the next generation of functional beverages.

## 5. Conclusions

This investigation demonstrated that the fermentation of an oat base with a starter strain of *Lactobacillus delbrueckii* subsp. *bulgaricus*, along with preliminary enzymatic hydrolysis, homogenization and pectin addition, considerably enhanced the physicochemical, rheological, antioxidant and sensory properties of the end fermented oat product. The addition of pectin resulted in a number of changes to the fermented oat beverages, including an increase in viscosity, texture and moisture retention capacity. It also led to an improvement in taste perception by enhancing flavor and consistency. The process of homogenization led to enhanced stability within the matrix and the capacity for extracting bioactive components.

The samples’ titratable acidity, phenolic content and antioxidant potential (RSA, OH-SA, and FRAP) increased during fermentation, especially in the presence of pectin. High radical-binding and enzyme inhibitory activities (lipase and glucosidase) were also shown by extractable polysaccharides from the fermented oat base, indicating a potent metabolic benefit. In terms of structural integrity, functionality and consumer acceptability, the combination of homogenization and pectin addition (Homog+) gave the best overall results.

These results highlight the potential of combining lactic acid fermentation with physical and compositional modifications to produce stable, palatable, nutrient-rich plant-based beverages. This approach could form the basis for developing new health-conscious, vegetarian and lactose-intolerant-friendly functionalized non-dairy fermented products.

## Figures and Tables

**Table 1:** The description of samples.

Sample	Starter Culture: *L. delbrueckii* subsp. *bulgaricus*	Pectin Content	Short Description
Control group			
Control	No	No	The crushed 1:4 mixture oat flakes and water treated by amylolytic enzymes. Not fermented by *L. bulgaricus*.
Control+	No	Yes	The crushed 1:4 mixture oat flakes and water treated by amylolytic enzymes. Add. pectin (1%). Not fermented by *L. bulgaricus*.
Control_h	No	No	The “control” additionally milled by the homogenizer. Not fermented by *L. bulgaricus*.
Control_h+	No	Yes	The “Control_h” additionally milled by the homogenizer. Add. pectin (1%). Not fermented by *L. bulgaricus*.
Experimental group			
Unhomog	Yes	No	The “Control” fermented by *L. bulgaricus*.
Unhomog+	Yes	Yes	The “Control+” fermented by *L. bulgaricus*.
Homog	Yes	No	The “Control_h” fermented by *L. bulgaricus*.
Homog+	Yes	Yes	The “Control_h+” fermented by *L. bulgaricus*.

**Table 2:** The indicators of sensory characteristic hedonic scale.

Indicator	Short Description	Significance Coefficient, k
Appearance	A characteristic of a product formed by sight in transmitted and reflected light during mixing and pouring (if necessary). It is necessary to exclude the other senses as much as possible.	0.10
Color	A characteristic of the color or color scheme of a product formed by a visual evaluation of the product.	0.10
Consistency	A set of rheological characteristics of products perceived by mechanical and tactile receptors. It is reasonable to understand consistency as a characteristic of the mobility (density) of viscous liquids.	0.15
Texture	A set of mechanical, geometric and surface characteristics of a product that are perceived by mechanical, tactile and, where possible, visual and auditory receptors. Texture is perceived tactilely in the oral cavity when consuming the product with the involvement of elements of the mechanical impact on the product from the teeth, tongue, and palate (pressing, crushing, and chewing). It forms the “body” of the product.	0.20
Taste	The presence of flavor, per se, perceived by the receptors initially upon contact with the product.	0.10
Aftertaste	A set of residual receptor responses after exposure to a product on the oral cavity, tongue, and palate.	0.05
Smell	The presence of an odor, per se, perceived by the receptors initially upon contact with the product.	0.10
Flavor	The totality of all the elements that form the overall perception of aroma-forming sensations by the senses of touch.	0.05
General acceptability	The overall assessment of consumer properties of the product according to the totality of all indicators.	0.15

**Table 3:** The matrix for evaluating the sensory profile.

Indicator	Evaluation Criteria				
	1	2	3	4	5
Appearance	unsatisfactory	poor	satisfactory	good	excellent
Consistency	not thickened	not thickened	thickened	viscous, homogeneous	thick, homogeneous
Color	neutral	neutral	typical of this raw material	pleasant, with a beige shade	pleasant, pronounced
Texture	watery	watery	light, not watery	elastic, velvety	elastic, gummy, enveloping
Taste	not pronounced	weak	weakly pronounced	pronounced	pleasant, pronounced
Aftertaste	insignificant	insignificant	significant	developed	long aftertaste, harmonious
Smell	neutral	weak	pleasant	pronounced, typical	pronounced, strong
Flavor	not pronounced	weak	significant	developed	intense, balanced
General Acceptability	dislike extremely	dislike	like moderately	like very much	like extremely

**Table 4:** Parameters of the oat base before and after hydrolysis.

Parameter	Before	After
pH	7.04 ± 0.03	6.52 ± 0.03
Titrable acidity, °T	8.5 ± 0.05	14.0 ± 0.06
Protein, %	2.16 ± 0.01	2.56 ± 0.01
Fat, %	0.99 ± 0.01	1.03 ± 0.01
Dry matter, %	12.57 ± 0.05	13.95 ± 0.08
Total sugar, %	2.73 ± 0.02	8.26 ± 0.05
Glucose, mmol/L	0.1 ± 0.01	12.8 ± 0.02

**Table 5:** Chemical parameters of *L. bulgaricus*-fermented oat base.

Sample	Protein, %		Fat, %		Dry Matter, %	
	No Pectin	+Pectin	No Pectin	+Pectin	No Pectin	+Pectin
Control	1.95 ± 0.05	2.19 ± 0.05 ^ab^	0.93 ± 0.03	1.03 ± 0.01	12.16 ± 0.22	13.64 ± 0.24 ^a^
Control_h	1.94 ± 0.06	2.27 ± 0.06 ^a^	0.95 ± 0.01	1.10 ± 0.02 ^ab^	13.14 ± 0.21 ^b^	13.38 ± 0.22 ^a^
Unhomog	1.93 ± 0.06	2.24 ± 0.06 ^a^	0.91 ± 0.01	1.10 ± 0.02 ^ab^	12.74 ± 0.21 ^b^	13.43 ± 0.22 ^a^
Homog	1.85 ± 0.06	2.16 ± 0.07 ^a^	0.90 ± 0.02	1.11 ± 0.02 ^ab^	12.16 ± 0.23	14.39 ± 0.24 ^ab^

^a^ indicates statistically significant differences between variants without pectin and with pectin according to non-parametric one-way analysis of variance (Kruskal–Wallis) test, *p* < 0.05. ^b^ indicates statistically significant differences between oat_base (oat_base + pectin) and fermented beverages with non-parametric one-way analysis of variance (Kruskal–Wallis) test, *p* < 0.05.

**Table 6:** Structure and stability properties of *L. bulgaricus*-fermented oat base.

Sample	Apparent Viscosity, cP		L_ƞ_, %		CMR	
	No Pectin	+Pectin	No Pectin	+Pectin	No Pectin	+Pectin
Control	ND	114.9 ± 2.0 ^ab^	-	14.2 ^a^	-	0.88 ^a^
Control_h	ND	180.3 ± 2.0 ^ab^	-	11.3 ^a^	-	0.92 ^a^
Unhomog	165.2 ± 1.3 ^a^	285.1 ± 2.2 ^ab^	2.9 ^b^	1.5 ^b^	0.98	0.98 ^b^
Homog	283.0 ± 3.4 ^a^	376.1 ± 7.3 ^ab^	1.04 ^b^	1.0 ^b^	0.99	0.99 ^b^
**Sample**	**Syn, %**		**Δ Syn, % to No Pectin**	**WHC, %**		**Δ WHC, % to No Pectin**
	**No Pectin**	**+Pectin**		**No Pectin**	**+Pectin**	
Control	52.9 ± 2.3 ^ab^	11.0 ± 1.4 ^ab^	−79.2	30.3 ± 1.2 ^ab^	55.2 ± 1.6 ^ab^	+82.2
Control_h	45.4 ± 2.3 ^ab^	9.0 ± 1.4 ^ab^	−80.2	36.3 ± 1.2 ^ab^	58.2 ± 1.6 ^ab^	+60.3
Unhomog	24.3 ± 1.8 ^ab^	1.5 ± 0.7 ^ab^	−96.0	47.5 ± 1.4 ^ab^	98.0 ± 1.0 ^ab^	+108.6
Homog	3.1 ± 0.6 ^ab^	0.5 ± 0.1 ^ab^	−84.1	93.0 ± 1.4 ^ab^	98.0 ± 1.0 ^ab^	+7.0

^a^ indicates statistically significant differences between variants without pectin and with pectin according to non-parametric one-way analysis of variance (Kruskal–Wallis) test, *p* < 0.05. ^b^ indicates statistically significant differences between oat_base (oat_base + pectin) and fermented beverages to non-parametric one-way analysis of variance (Kruskal–Wallis) test, *p* < 0.05.

**Table 7:** The texture parameters of *L. bulgaricus*-fermented oat base.

Sample	Hardness, g		Cohesiveness, %	
	No Pectin	+Pectin	No Pectin	+Pectin
Control	11.22 ± 0.56	12.11 ± 0.61	94.34 ± 4.72	97.61 ± 4.88
Control_h	11.58 ± 0.56	12.47 ± 0.61	96.34 ± 4.82	98.15 ± 4.91
Unhomog	12.53 ± 0.63	13.21 ± 0.69	75.90 ± 3.80	81.51 ± 4.08
Homog	11.92 ± 0.60	13.12 ± 0.66 ^a^	92.68 ± 4.63	96.37 ± 4.92
**Sample**	**Gumminess, g**		**Adhesiveness, g·mm**	
	**No Pectin**	**+Pectin**	**No Pectin**	**+Pectin**
Control	10.60 ± 0.53	11.81 ± 0.59 ^a^	45.44 ± 2.27	51.53 ± 2.58 ^a^
Control_h	11.15 ± 0.56	11.91 ± 0.59 ^a^	44.24 ± 2.21	52.49 ± 2.62 ^a^
Unhomog	9.5 ± 0.48 ^b^	11.16 ± 0.56 ^a^	52.2 ± 2.61 ^b^	56.85 ± 2.84 ^a^
Homog	11.72 ± 0.59 ^b^	12.12 ± 0.61 ^a^	49.63 ± 2.48 ^b^	54.86 ± 2.74 ^a^

^a^ indicates statistically significant differences between variants without pectin (control) and with pectin according to non-parametric one-way analysis of variance (Kruskal–Wallis) test, *p* < 0.05. ^b^ indicates statistically significant differences between oat_base (oat_base + pectin) and fermented beverages with non-parametric one-way analysis of variance (Kruskal–Wallis) test, *p* < 0.05.

**Table 8:** Scoring results of sensory profile *L. bulgaricus*-fermented oat base.

Indicator	Sample		Mean	
Appearance	Unhomog		0.40 ± 0.02	
	Homog		0.40 ± 0.02	
	Unhomog+		0.45 ± 0.02 ^ab^	
	Homog+		0.48 ± 0.02 ^ab^	
Color	Unhomog		0.41 ± 0.02	
	Homog		0.42 ± 0.02	
	Unhomog+		0.43 ± 0.02	
	Homog+		0.43 ± 0.02	
Consistency	Unhomog		0.51 ± 0.03 ^ab^	
	Homog		0.63 ± 0.03 ^ab^	
	Unhomog+		0.68 ± 0.03 ^ab^	
	Homog+		0.74 ± 0.04 ^ab^	
Texture	Unhomog		0.64 ± 0.04 ^ab^	
	Homog		0.82 ± 0.04 ^ab^	
	Unhomog+		0.92 ± 0.05 ^ab^	
	Homog+		0.98 ± 0.05 ^ab^	
Taste	Unhomog		0.42 ± 0.02 ^ab^	
	Homog		0.45 ± 0.02 ^ab^	
	Unhomog+		0.47 ± 0.02 ^ab^	
	Homog+		0.49 ± 0.02 ^ab^	
Aftertaste	Unhomog		0.11 ± 0.01 ^ab^	
	Homog		0.17 ± 0.01 ^ab^	
	Unhomog+		0.21 ± 0.01 ^ab^	
	Homog+		0.24 ± 0.01 ^ab^	
Smell	Unhomog		0.31 ± 0.02	
	Homog		0.31 ± 0.02	
	Unhomog+		0.43 ± 0.02 ^ab^	
	Homog+		0.45 ± 0.02 ^ab^	
Flavor	Unhomog		0.14 ± 0.01	
	Homog		0.16 ± 0.01	
	Unhomog+		0.22 ± 0.01 ^ab^	
	Homog+		0.23 ± 0.01 ^ab^	
General acceptability	Unhomog		0.50 ± 0.03 ^ab^	
	Homog		0.57 ± 0.03 ^ab^	
	Unhomog+		0.67 ± 0.03 ^ab^	
	Homog+		0.70 ± 0.03 ^ab^	
Total	Unhomog	Homog	Unhomog+	Homog+
	3.4	3.9	4.5	4.7

^a^ indicates statistically significant differences between variants without pectin and with pectin according to non-parametric one-way analysis of variance (Kruskal–Wallis) test, *p* < 0.05. ^b^ indicates statistically significant differences between fermented beverages to non-parametric one-way analysis of variance (Kruskal–Wallis) test, *p* < 0.05.

## Data Availability

The original contributions presented in the study are included in the article, further inquiries can be directed to the corresponding author.
